# Interleukin 35: A Key Mediator of Suppression and the Propagation of Infectious Tolerance

**DOI:** 10.3389/fimmu.2013.00315

**Published:** 2013-10-18

**Authors:** Brian M. Olson, Jeremy A. Sullivan, William J. Burlingham

**Affiliations:** ^1^Department of Medicine, Carbone Cancer Center, University of Wisconsin, Madison, WI, USA; ^2^Department of Surgery, University of Wisconsin Hospital and Clinics, Madison, WI, USA

**Keywords:** interleukin 35, infectious tolerance, natural regulatory T cells, induced regulatory T cells, iTr35

## Abstract

The importance of regulatory T cells (Tregs) in balancing the effector arm of the immune system is well documented, playing a central role in preventing autoimmunity, facilitating graft tolerance following organ transplantation, and having a detrimental impact on the development of anti-tumor immunity. These regulatory responses use a variety of mechanisms to mediate suppression, including soluble factors. While IL-10 and TGF-β are the most commonly studied immunosuppressive cytokines, the recently identified IL-35 has been shown to have potent suppressive function *in vitro* and *in vivo*. Furthermore, not only does IL-35 have the ability to directly suppress effector T cell responses, it is also able to expand regulatory responses by propagating infectious tolerance and generating a potent population of IL-35-expressing inducible Tregs. In this review, we summarize research characterizing the structure and function of IL-35, examine its role in disease, and discuss how it can contribute to the induction of a distinct population of inducible Tregs.

## Introduction

The immune system has evolved to establish multiple layers of defense against a variety of pathogens and diseases. However, concurrent with these effector responses are a robust network of regulatory responses that are able to keep the effector branch of the immune system in check and ensure that they do not lead to potentially harmful autoimmunity. These suppressive responses are mediated by a myriad of cell types, including myeloid-derived suppressor cells as well as macrophages with suppressive function [such as tumor-associated macrophages (1)], but suppressive function is most commonly associated with regulatory T cells (Tregs). T cells with potential suppressive activity were identified in the seminal research by Gershon and Kondo as well as Nishizuka and Sakakura more than 40 years ago, showing that lymphocytes can suppress T cell responses and that this tolerance could be transferred into naive mice (2–5). However, after this foundational work, research into Tregs went through a period of controversy and conflicting results, with difficulty in identifying a molecular basis for their suppressive function leading some to question their existence. Following more than a decade of studies aimed at elucidating the mechanisms that mediate Treg activity, interest was rekindled in the mid-1990s with the transformational research of Sakaguchi and colleagues, who specifically identified a population of CD4+CD25+ T cells that had suppressive function, which were coined as naturally occurring thymic-derived Tregs, or natural Tregs (nTreg) (6). These Tregs were later identified as also expressing the intracellular transcription factor Foxp3, and were found to mediate suppression against a wide array of effector immune responses, including CD4+ and CD8+ T cells, B cells, natural killer (NK), and NK-T cells, and even inducing dendritic cells (DCs) and macrophages into a more tolerogenic phenotype. However, while nTregs play a central role in mediating tolerance to a variety of self antigens, they are recognized as not being the primary mediator of tolerance to pathogens and other antigens encountered in the periphery. This role belongs to a broad class of cells classified as peripherally derived or inducible Tregs (iTregs), which are CD4+ or CD8+ T cells which enter the periphery as naive T cells but encounter their antigen under conditions which are not conducive to the generation of productive effector responses, such as environments rich in immunosuppressive cytokines such as TGF-β. When activated in these conditions, iTregs gain potent suppressive functions, inhibiting T-cell proliferation and effector functions in an antigen non-specific fashion, and play a central role in mediating regulation and propagating infectious tolerance in a variety of malignancies, including infectious diseases and cancer.

Inducible Tregs are further divided into subclasses of iTregs, which are classified based largely on the mechanisms they use to mediate regulation (though the functional mechanisms of suppression utilized by these various iTregs are not strictly limited to each subpopulation – for example, multiple iTreg populations use surface molecules such as CTLA-4 or PD-1 to mediate infectious tolerance). Tr1 induced regulatory cells mediate suppression primarily through the secretion of the immunosuppressive cytokine IL-10, and are further characterized by their lack of Foxp3 and CD25 expression which are expressed by nTregs (7). The second class of iTregs are Th3 cells, which were one of the earliest populations of Tregs and were identified as playing a role in mediating tolerance in experimental autoimmune encephalitis (EAE). These cells express CD25 and Foxp3 and predominantly utilize TGF-β to mediate suppression, with minimal expression of IL-4 and IL-10 (8).

While the Tr1 and Th3 populations of iTregs were long considered to be the only defined induced regulatory populations, research has identified another population of induced Tregs that can are potent mediators of suppression as well as in the propagation of infectious tolerance: iTr35 regulatory cells. These inducible regulatory cells were identified by Dario Vignali and colleagues and mediate suppression primarily through the expression of the regulatory cytokine IL-35 (9). In this review, we will discuss the identification and characterization of IL-35, how it mediates suppression, the role it has been shown to play in disease, and the importance of IL-35 and more specifically iTr35 cells in propagating infectious tolerance.

## IL-35: Composition, Signaling, and Expression

**Figure 1:**
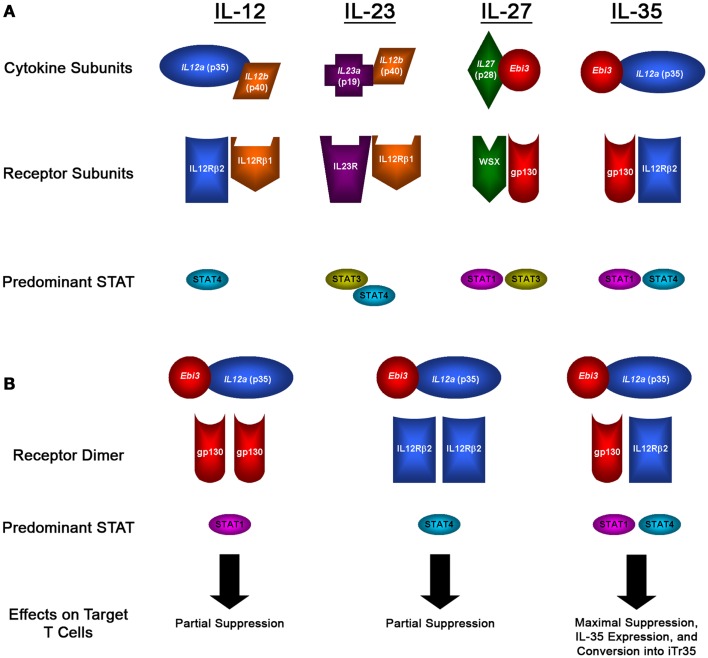
**IL-12 family members and signaling pathways**. **(A)** Diagram showing the subunits that form the heterodimeric IL-12 family of cytokines, the subunits that form their receptors, and the predominant STAT molecules that transmit their signals. **(B)** Diagram showing the potential receptor and signaling pathways utilized by IL-35, which can signal through gp130 or IL-12Rβ2 homodimers, or through a unique gp130:IL-12Rβ2 heterodimer, which results in the formation of a novel STAT1:STAT4 heterodimer that has distinct effects on target cells: maximal suppression, IL-35 expression, and their conversion into iTr35 regulatory T cells.

Interleukin 35 belongs to the IL-12 family of cytokines, which is a group of heterodimeric cytokines that are composed of one of five subunits [p19, p28, p35, p40, and Epstein–Barr virus-induced gene 3 (Ebi3)] that come together in various combinations to form IL-12, IL-23, IL-27, and IL-35, as illustrated in Figure 1A (10). Despite their shared components, these cytokines run the spectrum of immunological effector functions. IL-12 is a pro-inflammatory cytokine that is closely associated with the activation of Th1 immune responses. It is predominantly expressed by monocytes and DCs, and its expression can be triggered by activated T cells. The inflammatory activity of IL-12 is clearly seen in efforts to target its activity in a variety of diseases. In patients with malignancy, research has shown that recombinant IL-12 can elicit anti-tumor responses *in vivo* (11). Alternatively, efforts to inhibit the inflammatory effects of IL-12 have been developed, including IL-12-blocking antibodies used to treat autoimmune disorders such as EAE, where it has been shown to prevent uncontrolled immune responses (12, 13). Similar to IL-12, IL-23 also has inflammatory activity and can drive Th1 responses, as well as promoting the activity of NK and Th17 cells (14). As opposed to IL-12 and IL-23, IL-27 has a wide range of immunomodulatory activities. While it can promote Th1 development, IL-27 can also inhibit Th2 responses and promote the suppression of T-cell responses depending on the microenvironment (15).

While IL-12, IL-23, and IL-27 can all play a role in promoting inflammatory immune responses, the youngest member of the IL-12 family, IL-35, is a purely immunosuppressive cytokine. IL-35 was identified in the mid-2000s, first reported by Dario Vignali and colleagues, and was soon after reported by his group and others to be a potent mediator of suppression (9, 16). IL-35 is a heterodimer composed of the p35 and Ebi3 subunits, which were both identified as being over-expressed by Tregs and not effector cells (9). The potential of these two subunits coming together to form a novel heterodimer was first described in 1997 by Devergne and colleagues, who found that cells transfected with p35 and Ebi3 lead to the secretion of a p35-Ebi3 heterodimer (17). In this report, it was suggested that given the expression of Ebi3 in many tissues replete with immune cells, it was likely that this heterodimer had immunomodulatory function – however, no functional studies were conducted for another 10 years. Recent studies into the formation of this heterodimer found that subunits from human and mouse can bind to the opposite species, indicating that the protein-protein interactions that form IL-35 are novel to the IL-12 family and conserved between species (18). Furthermore, the protein binding sites were unique when compared to those used for IL-12 and IL-27, and that no single mutation could disrupt this interaction (18). This is particularly significant, as the design of therapeutic interventions aimed at targeting the suppressive activity of IL-35 could focus on small-molecule inhibitors of this interaction which would selectively target IL-35 while leaving IL-12 and IL-27 unaffected.

In addition to having a unique function when compared to the other IL-12 family members, IL-35 is also unique in that rather than being expressed primarily by antigen-presenting cells (APCs), IL-35 is expressed primarily by Tregs. Since it was identified in 2007, dozens of reports have been published describing IL-35 expression in both thymus-derived and peripheral Tregs. This includes a subset of CD4+CD25+Foxp3+ nTregs in humans, mice, and even pigs (9, 19, 20), though this expression is thought to occur only in a subset of IL-35+ nTregs and is not constitutive (21). Research has also identified expression of IL-35 in a population of IL-35-induced CD4+ Tregs, defined as iTr35 cells (22). In addition to CD4+ Tregs, IL-35 has also been shown to be expressed and mediate antigen-specific suppression in a population of CD8+ Tregs in patients with prostate cancer (23). Interestingly, other non-immune cell types have also been shown to express IL-35, including tumor cells (24, 25) and potentially an even broader tissue expression profile in the course of inflammation (26). However, in all of these cell types, it has been noted that IL-35 expression is minimal in unactivated T cells – rather, these cells need to become activated for the induction of IL-35, such as through engagement of their T-cell receptor or following inflammation (19, 22, 26). This suggests that IL-35 may be more associated with the suppressive activity of Tregs in peripheral tissues rather than a constitutive marker of Tregs. The suggested expression of IL-35 by multiple cells types, including both natural and induced Treg, emphasizes the need to further characterize the mechanisms that regulate the expression of IL-35 in these populations.

After being expressed and secreted by Tregs, IL-35 then acts on its target cells following binding to the IL-35 receptor. However, as is the case with the subunits that make up the IL-12 family of heterodimeric cytokines, the receptors for the IL-12 family are also composed of five different subunits: IL-12Rβ1, IL-12Rβ2, IL-23R, WSX, and gp130 (as illustrated in Figure 1B). The IL-35 receptor is composed of IL-12Rβ2 and gp130, which are also associated with the IL-12 and IL-27 receptors, respectively (27, 28). Following binding of IL-35 to its receptor, its signal is transduced through STAT1 and STAT4, which can also form a unique heterodimer and result in the expression of target genes including p35 and Ebi3, resulting in a feedback loop promoting increased IL-35 expression (28). However, IL-35 is also unique from the other members of the IL-12 family in that it can also signal through a homodimer of its receptor subunits. However, when IL-35 binds to one of its homodimeric receptors, only one branch of its signal transduction pathway is activated (either STAT1 or STAT4 for gp130:gp130 or IL-12Rβ2:IL-12Rβ2 homodimers, respectively), resulting in a partial loss of the suppressive activity of IL-35 compared with signaling through the fully functional IL-12Rβ2-gp130 heterodimer receptor, as diagramed in Figure 1B (28). The use of these subunits sheds some light onto the target of IL-35 activity; gp130 is expressed in most cell types (29), whereas IL-12Rβ2 is expressed predominantly on activated T cells, NK cells, and to a lesser extent DCs and B cells (30).

## IL-35: Mechanisms of Suppression and Role in Disease

Since its discovery, the predominant mechanism of suppression associated with the activity of IL-35 is its ability to suppress T-cell proliferation and effector functions. Foundational work into the activity of IL-35 utilized *IL-12a*^−/−^ and *Ebi3*^−/−^ mice, finding that CD4+ Treg lacking IL-35 expression had a significantly reduced ability to suppress T-cell proliferation (9), an observation that has been repeated in numerous models by several groups (19, 22, 31–33). The ability of IL-35 to suppress T-cell responses has also been clearly illustrated in studies using recombinant IL-35 (rIL-35), where it can decrease T-cell proliferation as well as T-cell cytokine expression, though these studies have been somewhat complicated by the difficulty in generating an active heterodimeric form of IL-35 (9, 16, 34, 35). In related studies, the ectopic expression of IL-35 by conventional CD4+ T cells (using a transfected IL-35 expression construct) results in these conventional T cells gaining a regulatory phenotype, manifested by the ability to potently suppress T-cell proliferation (9, 22). The suppressive activity of IL-35 is not limited to CD4+ Tregs, as a population of CD8+CTLA-4+ Tregs was also found to suppress the proliferation of autologous T cells in a contact-independent, IL-35-dependent fashion (23).

While mechanistic studies into IL-35 have focused on its ability to suppress CD4+ and CD8+ T-cell proliferation, IL-35 has also been shown to have a role in suppressing Th17 responses. Tregs expressing IL-35 have been shown to inhibit the differentiation of CD4+ T cells into Th17 effector cells, and mice lacking Ebi3 have a significant increase in the production of IL-17 (32, 36–38). This has also been reproduced in studies using rIL-35, in which treatment with rIL-35 reduces Th17 differentiation as well as the function of Th17 cells (16, 34). In addition to its effects on Th17 immunity, one report has even suggested that rIL-35 can lead to decreased antibody titers (34). While this is the only report to our knowledge to associate IL-35 activity with the suppression of humoral immunity, it has significant implications toward the precise mechanism of action of IL-35. While *in vitro* studies have clearly shown that IL-35 is able to directly act on effector T cells (supported by the expression profiles of the IL-35 receptor subunits), the ability of IL-35 to suppress antibody responses could suggest that IL-35 is also able to act on other cell populations, though it could also be a reflection of the inhibition of helper T cell responses that contribute to humoral immunity.

Given the direct suppressive activity of IL-35, there has been interest in evaluating the role that IL-35 can play in the development of a variety of diseases (summarized in Table 1). Several diseases have been shown to be associated with increased IL-35 expression, including multiple inflammatory diseases, coronary artery disease, and cancer. In individuals with acute myeloid leukemia, the development of disease has been shown to be associated with elevated plasma levels of IL-35 (39). This has been supported by results in lung cancer, where a study evaluating Ebi3 levels in lung cancer patients found that Ebi3 levels are elevated in patients with malignancy, predicts for poor outcome, and is an independent prognostic indicator of disease (although this study only examined Ebi3, and not the p35 subunit of IL-35) (40). Additionally, in murine models of melanoma and colorectal carcinoma, the establishment of tumors has been shown to lead to increased IL-35 expression in CD4+ tumor-infiltrating lymphocytes, which are subsequently able to suppress T-cell proliferation (22). This likely contributes to the inhibition of the anti-tumor effects of adoptively transferred CD4+ and CD8+ T cells in this melanoma model.

**Table 1:** **Role of IL-35 on disease**.

Disease/model	Method of IL-35 detection	Effects of disease on IL-35 expression	Reference
**STUDIES EVALUATING EFFECTS OF DISEASE ON IL-35 EXPRESSION**			
Acute myeloid leukemia	ELISA	Patients with AML have significantly higher plasma levels of IL-35 than healthy donors	Wu et al. (39)
Allergic airway disease	qPCR	Induction of allergic airway disease leads to increased Treg that express IL-35	Whitehead et al. (37)
Colorectal carcinoma	qRTPCR	Tumor injections lead to increase in IL-35 expression in tumor-infiltrating CD4+ T cells (CD4+Foxp3+ and CD4+Foxp3−)	Collison et al. (22)
Coronary artery disease	ELISA	Decreased IL-35 correlates with increased left ventricular ejection fraction	Lin et al. (41)
Lung cancer	Immunohistochemistry and ELISA	Lung cancer patients have significantly elevated serum levels of Ebi3, and elevated Ebi3 expression correlates with poor prognosis, and is an independent prognostic factor of disease	Nishino et al. (40)
Melanoma	qRTPCR, Western blot	Tumor injections lead to increase in IL-35 expression in tumor-infiltrating CD4+ T cells (CD4+Foxp3+ and CD4+Foxp3−), which can suppress T-cell proliferation	Collison et al. (22)
Smoking-induced lung inflammation	ELISA	Animals exposed to cigarette smoke and treated with erythromycin have increased levels of IL-35 in bronchoalveolar lavage fluid	Bai et al. (42)
*Trichuris muris* infection	qRTPCR	Infection with *Trichuris muris* induces significant increase in IL-35 expression in intestinal Tregs	Collison et al. (22)

**Disease/model**	**Mechanism of IL-35 expression**	**Effects of IL-35 expression on disease**	**Reference**

**STUDIES EVALUATING EFFECTS OF IL-35 ON DISEASE**			
Allergic airway disease	Gene therapy using plasmid DNA encoding single-chain IL-35 fusion protein	IL-35 gene therapy decreases allergic airway inflammation and inflammation-associated antibody responses	Huang et al. (43)
Autoimmune diabetes	Ectopic expression of rIL-35 in non-obese diabetic (NOD) mice	IL-35 expression protects animals from autoimmune diabetes by a decrease in T-cell infiltration and proliferation (via G1 arrest)	Bettini et al. (44)
Cancer	Ectopic expression of IL-35 in murine tumor cell lines	IL-35 expression increases tumorigenesis by increasing infiltration of CD11b+Gr1+ myeloid cells and thus increasing tumor angiogenesis, as well as a decrease in the numbers and effector functions of CD4+ and CD8+ TIL	Wang et al. (25)
Collagen-induced arthritis (CIA)	Intraperitoneal injection of single-chain rIL-35	IL-35 reduces incidence, intensity, and progression of CIA, a reduction of CIA-specific antibodies, a reduction of Th1 and Th17, and protective CD4+CD39+CD25−Tregs	Kochetkova et al. (34)
Collagen-induced arthritis (CIA)	Intraperitoneal injection of rIL-35	IL-35 induces a significant reduction in the incidence and intensity of CIA	Niedbala et al. (16)
Inflammatory bowel disease	Adoptive transfer of iTr35 cells into IBD-bearing *Rag1*^−/−^mice	iTr35 cells cure inflammatory bowel disease	Collison et al. (22)
Inflammatory bowel disease	Gene therapy using plasmid DNA encoding single-chain IL-35 fusion protein	IL-35 gene therapy decreases symptoms of colitis and decrease in colonic inflammatory markers	Wirtz et al. (35)
Lyme arthritis	Subcutaneous injection of rIL-35	rIL-35 enhances Lyme arthritis in *Borrelia*-infected and -vaccinated mice	Kuo et al. (45)
Melanoma	Adoptive transfer of iTr35 cells into tumor-bearing *Rag1*^−/−^mice	iTr35 cells suppress anti-tumor responses generated following adoptive transfer of CD4+ and CD8+ T cells	Collison et al. (22)

**Disease/model**	**Transgenic mouse model**	**Effects of knock-out on disease**	**Reference**

**STUDIES EVALUATING EFFECTS OF IL-35 KNOCK-OUT ON DISEASE**			
Allergic airway disease	*Ebi3*^−/−^mice	Ebi3 is required for control of airway inflammation	Whitehead et al. (37)
Coronavirus-induced encephalomyelitis	*Ebi3*^−/−^mice	*Ebi3*^−/−^mice have an increased viral load and increased mortality, increased T cell and macrophage infiltration, and enhanced viral-specific T-cell responses	Tirotta et al. (33)
Experimental autoimmune encephalomyelitis (EAE)	*Ebi3*^−/−^mice	nTreg or iTr35 cells, but not iT_R_con or *Ebi3*^−/−^iTr35 cells, prevent severity of EAE	Collison et al. (22)
Experimental autoimmune encephalomyelitis (EAE)	*Ebi3*^−/−^mice	*Ebi3*^−/−^mice have marginally increased EAE, and significantly increased Th1 and Th17 responses	Liu et al. (32)
Homeostatic expansion	*Ebi3*^−/−^mice	Adoptive transfer of iTr35, but not iTr35 cells lacking IL-35 expression (*Ebi3*^−/−^mice or WT mice given IL-35 blocking antibody), cells can prevent homeostatic expansion	Collison et al. (22)
Inflammatory bowel disease	*IL-27p28*^−/−^and *Ebi3*^−/−^mice	Ebi3-deficient mice have increased colitis, shorter survival, and increased expression of inflammatory markers (not seen in *IL-27p28*-deficient mice)	Wirtz et al. (35)
Inflammatory bowel disease	*Ebi3*^−/−^and *IL-12a*^−/−^mice	Transfer of Treg cures inflammatory bowel disease, but not Treg that lack either Ebi3 or IL-12a	Collison et al. (9)
Lethal autoimmunity	*Ebi3*^−/−^and *IL-12a*^−/−^mice	Adoptive transfer of nTreg or iTr35 cells can prevent lethal autoimmunity, but iTr35 cells lacking Ebi3 or IL-12a could not prevent autoimmunity	Collison et al. (22)
Liver fibrosis	*IL-12p35*^−/−^and *IL-12p40*^−/−^mice	*IL-12p35*^−/−^mice (but not *IL-12p40*^−/−^mice) have increased liver inflammation, bile duct damage, and development of Th17 responses	Tsuda et al. (38)

The loss of IL-35 has also been shown to be associated with the development and exacerbation of disease, including many inflammatory diseases such as encephalomyelitis and inflammatory bowel disease (Table 1). In multiple models of encephalomyelitis, wild-type Tregs can prevent the onset and severity of disease. However, animals that lack functional IL-35 were shown to have enhanced inflammatory immune responses and increased disease (9, 32, 33). Similar observations have been shown in inflammatory bowel disease, liver fibrosis, and models of lethal autoimmune disease (9, 22, 35, 38). Conversely, given that the loss of IL-35 is associated with increased incidence and severity of inflammatory diseases, the induction of IL-35 expression has been shown to alleviate a variety of disease symptoms (Table 1). In models of inflammatory bowel disease, IL-35 gene therapy and the adoptive transfer of IL-35-expressing Tregs have been shown to cure colitis symptoms (22, 35). The same holds true in collagen-induced arthritis, where rIL-35 reduces the frequency and severity of arthritis and a decrease in inflammatory immune responses (16, 34). As opposed to these inflammatory diseases, tumor models have shown that IL-35 contributes to tumorigenesis (22, 25). These effects are mediated through both immune-directed and tumor-directed effects, as IL-35 can act to suppress tumor-infiltrating lymphocytes that may have anti-tumor activity, as well as potentially supporting the proliferation of tumor cells by promoting angiogenesis (22, 25).

While the direct suppressive activity of IL-35 has been established in numerous reports *in vitro* and *in vivo*, research into immune tolerance has shown that the low frequency of individual regulatory populations alone are largely insufficient to control effector immunity. Therefore, to expand the breadth of suppressive immunity, Tregs are able to induce and mobilize additional regulatory immune cells. This concept of infectious tolerance is central to the ability of the immune system to maintain tight control of effector responses, whereby Tregs can transfer suppressive function onto a nominally conventional T cell population. Suppressive cytokines play a central role in the propagation of infectious tolerance, including IL-35, which has been shown to play an important role in the expansion of regulatory immunity.

## Role of IL-35 in Propagating Infectious Tolerance

The concept of infectious tolerance was first proposed by Gershon and Kondo in the early 1970s, where they showed that, “tolerance … can be spread from one cell to another” (4). This was further elucidated by Benjamin and Waldmann, who used antibodies blocking T cell populations to induce tolerance to skin grafts (46), and later in elegant studies by Qin and colleagues from the same group, who used congenically marked T cells to show that suppressive activity can be transferred from one cell population to another (47). As additional molecular data regarding the suppressive mechanisms of Treg has become available, it has become clear that Tregs can secrete cytokines that can induce naïve and even effector T cells to gain a regulatory phenotype. This can occur by directly targeting effector T cells and causing them to gain a suppressive phenotype, as well as targeting DC populations and causing them to promote the conversion of effector cells into regulatory cells (48). The most commonly thought of cytokines involved in this conversion are Treg-produced IL-10 and TGF-β, which can drive the generation of Tr1 and Th3 cells, respectively. However, the ability to transmit infectious tolerance is also a characteristic of IL-35, the production of which can cause the conversion of conventional effector T cells into induced regulatory cells that are potent mediators of suppression *in vitro* and *in vivo*.

Some of the earliest reports of IL-35 began to shed light on the potential role of this cytokine in infectious tolerance. In a report by Niedbala and colleagues in 2007, they found that a rIL-35 fusion protein induced the proliferation of a population of CD4+CD25+Foxp3+ T cells, and which expressed IL-10 and suppressed T-cell proliferation *in vitro* (16). Additionally, in another report utilizing a recombinant single-chain IL-35, it was found that treatment of mice with rIL-35 resulted in a significant increase in IL-10 (but not TGF-β) production by CD4+ T cells in draining lymph nodes (34). When these mice were examined further for the impact of IL-35 on Treg function, they found that administration of IL-35 led to an increase in the frequency of CD4+CD39+ Tregs that expressed Foxp3 and IL-10, and that IL-35 promoted the proliferation of these T cells (34). Furthermore, when these CD4+CD39+ T cells were adoptively transferred they could protect animals from collagen-induced arthritis in an IL-10-dependent fashion (34). These data together suggest that IL-35 is able to promote the expansion of IL-10 producing iTregs, and that these Tregs are able to mediate suppression *in vitro* and *in vivo*.

While these studies provided the earliest evidence that IL-35 could play a role in the propagation of infectious tolerance, it remained unclear whether this induced regulatory population also expressed IL-35, or whether IL-35 played any role in mediating suppression in these induced regulatory cells. This was addressed in an expansive report from by Collison and colleagues in late 2010 that not only specifically studied the role that IL-35 plays in the conversion of conventional T cells into induced Tregs, but also evaluated the role that IL-35 has in mediating the suppressive function of these iTreg (22). In this report, they show that treating either human or mouse conventional CD4+Foxp3− T cells with IL-35 causes these *T*_conv_ to begin to express IL-35 (but not IL-10 nor TGF-β), and that these *T*_conv_ cells can then suppress T-cell proliferation in a contact-independent fashion. Further supporting the lack of a role for IL-10 and TGF-β, blocking either of these cytokines did not affect the suppressive function of these cells whereas blocking IL-35 significantly abrogated this suppression, suggesting that these IL-35-induced regulatory cells represented a novel population of iTregs rather than the conventional Tr1 or Th3 cells, which they defined as iTr35 cells.

The conversion of conventional T cells into an IL-35-expressing inducible Treg was also shown to occur when *T*_conv_ were cultured with nTreg, which have been shown to express higher levels of IL-35 when cultured with conventional T cells (49). When mouse nTreg were cultured with *T*_conv_ cells, the *T*_conv_ cells began to express IL-35 and were then able to suppress T-cell proliferation (22). Interestingly, this conversion was found to require IL-35 and IL-10 expression by nTregs; however, once these *T*_conv_ cells had gained a regulatory phenotype, IL-10 was not required for their suppressive activity. In a later report, the same group confirmed that this conversion of *T*_conv_ into IL-35-expressing iTregs occurred in a contact-independent fashion through the activity of IL-35, but did not require IL-10 nor TGF-β (19). Furthermore, in this report they also show that maximal Treg suppression requires not only IL-35 expression but also contact with *T*_conv_ that can subsequently be converted into iTr35 cells (19), further highlighting the importance of infectious tolerance in the overall suppressive activity of IL-35.

The generation of iTr35 cells has also been shown to occur naturally following the onset of various diseases. In one model, mice were infected with an intestinal parasite that induces an inflammatory response followed by the expansion of Treg responses in the intestine. Following this infection, CD4+ Foxp3+ nTregs in the spleen were found to have negligible levels of IL-35 expression, whereas CD4+Foxp3+ at the site of infection had a significant increase in IL-35 (22). Interestingly, when CD4+Foxp3− conventional T cells were examined for IL-35, negligible levels were found in the spleen, whereas CD4+Foxp3− T cells at the infection site had a significant increase in IL-35 expression (22). Similar results were observed in two different tumor models (MC38 colorectal and B16 melanoma tumor cell lines), where tumor inoculation led to an increase in IL-35 expression in CD4+Foxp3+ and CD4+Foxp3− T cells that infiltrated the tumor, whereas there was negligible IL-35 expression in splenic T cells (22). Furthermore, these tumor-infiltrating CD4+Foxp3− T cells were able to suppress T-cell proliferation *in vitro* in an IL-35-dependent fashion, indicating that tumor establishment led to the generation of iTr35 cells (22).

The observation that tumor formation leads to the generation of iTr35 cells suggests that these cells may play a role in promoting tumor development, a characteristic that is associated with other induced Treg populations. In a variety of malignancies, increased frequencies of Tregs has been shown to correlate with a poor prognosis for patients, though this observation is not absolute (50). The profoundly suppressive tumor microenvironment has been shown to promote the generation of regulatory immune responses, using factors such as TGF-β or adenosine to mediate the conversion of effector lymphocytes into iTregs (51, 52). Furthermore, these tumor-infiltrating iTreg have been shown to have greater suppressive activity that nTreg, both in terms of the levels of suppression as well as the mechanisms used (22, 53–55). However, this does not appear to be the case with IL-35, as tumor-infiltrating CD4+Foxp3+ nTregs had higher levels of suppression then infiltrating Foxp3− iTr35 cells (22). This likely reflects the multitude of suppressive mechanisms that nTreg are able to utilize to mediate suppression, as tumor-infiltrating *Ebi3*^−/−^ nTreg were able to suppression T-cell proliferation at equal levels compared with wild-type nTreg (22), and even Treg that lack both IL-35 and IL-10 expression can still mediate suppression through factors such as TRAIL (56). However, iTr35 cells appear to lack this functional plasticity, as *Ebi3*^−/−^ mice do not have tumor-infiltrating induced regulatory cells with suppressive function, and iTr35 cells lacking Ebi3 and/or IL-12p35 lack efficacy in preventing autoimmune responses in a variety of models in addition to these tumor models (22).

Despite this requirement for IL-35, iTr35 cells, and the role of IL-35 expression on the propagation of infectious tolerance is an important component of the suppressive tumor microenvironment. When *Rag1*^−/−^ mice are challenged with tumors and then receive adoptively transferred wild-type CD4+ and CD8+ T cells, these T cells are able to mediate an anti-tumor response and keep tumor growth in check (22). When wild-type Tregs are transferred as well, the tumors grow rapidly, reflecting the ability of Treg to suppress the anti-tumor response associated with the transfer of the CD4+ and CD8+ T cells, both by directly suppressing T-cell proliferation as well as converting these conventional T cells into regulatory cells (22). However, when tumor-bearing animals receive an adoptive transfer containing wild-type CD8+ T cells and *Ebi3*^−/−^ CD4+ T cells, the growth of these tumors was reduced by approximately 50% (22). This suggests that the conversion of *T*_conv_ into iTr35 cells is a significant contributor to the suppression of anti-tumor immunity, and that the therapeutic targeting of this regulatory population could promote anti-tumor responses.

The dependence of iTr35 cells on IL-35 also suggests that these cells may have different characteristics regarding their long-term phenotypic and functional stability. Given the nature of induced regulatory cells, which gain or lose immunosuppressive activity depending on the microenvironment in which they are activated, the stability of these induced populations is thought to be transient. However, data regarding the stability of iTr35 cells *in vivo* suggests otherwise. In numerous models, the transfer of iTreg was shown to mediate clinical efficacy for several weeks following a single adoptive transfer, suggesting that these cells retain their suppressive function for an extended period of time (22). Furthermore, when congenically marked iTr35 or Th3 cells were injected into mice and recovery was measured over time, it was found that 33% of the injected iTr35 cells were recoverable after 1 week, 30% after 2 weeks, and 20% after more than 3 weeks, compared with only 12% of Th3 cells that were recovered 1 week following transfer (22). Additionally, these cells retained their suppressive function; even 25 days following injection, adoptively transferred iTr35 cells were able to suppress T-cell proliferation at the same levels as freshly isolated iTr35 cells, whereas Th3 cells had significantly reduced suppressive function (22). This suggests that iTr35 cells may represent more of a terminally differentiated regulatory population, and rely on the potent suppressive activity of IL-35 to mediate suppression. However, further work is necessary to characterize these iTr35 cells, and other suppressive mechanisms that they may gain or lose over time.

## Concluding Remarks and Future Directions

As we look toward the future of immune regulation and infectious tolerance, it is essential to focus not only on identifying novel mediators of tolerance, but how these populations can be reliably identified. This is particularly relevant with the generation of induced Tregs during the propagation of infectious tolerance, as the plastic nature of these populations makes them challenging to identify and track over time. Even iTr35 cells, which may represent more of a stable regulatory population than Tr1 or Th3 cells, are incredibly challenging to identify. While these cells are predominantly identified based on their robust expression of IL-35, the detection of IL-35 expression can be daunting. The nature of the IL-12 family of cytokines requires the expression of all five subunits to be interrogated to ensure that it is IL-35 that is being expressed by a putative regulatory population (even though Tregs do not express significant levels of the other IL-12 family members). This is also manifested in difficulty detecting IL-35 protein levels using current commercially available reagents and the lack of an antibody that jointly recognizes a conformational epitope from IL-35, thus requiring Ebi3, p35, and the other IL-12 subunits to be examined. Additionally, the difficulty in generating functionally active rIL-35 also delayed research and led to the necessity of multiple levels of evaluation in studying the suppressive effects of IL-35. As these reagents are developed and become commercially available, it is to be hoped that increased research into IL-35 will better define and characterize Tregs that express this cytokine.

While IL-35 expression can be analyzed on fixed or lysed cell populations, what would be ideal would be a series of cell surface markers that can be used to identify iTr35 cells, allowing these cells to be isolated and further characterized. Currently, iTr35 cells are characterized only by their expression of CTLA-4 and CD25, as well as a lack of intracellular Foxp3; however, a gene microarray comparing iTr35 cells with conventional Treg found that there was no significant genetic signature that could be used to distinguish one regulatory population from the other (22). Furthermore, while treatment with rIL-35 was shown to induce a population of CD4+CD39+CD25−Tregs that express IL-10, these cells were not evaluated for expression of IL-35, causing CD39 to remain one of the potential markers of iTr35 cells, though its expression is clearly not restricted to iTr35 cells (34). This highlights the importance of elucidating markers for iTr35 cells that have not yet been evaluated [such as GITR, PD-1, CD127, or even HELIOS, whose expression is traditionally associated with nTreg but was recently suggested to also be present on iTreg (57, 58)] that can facilitate the identification of iTr35 cells without expression analysis.

As clearly illustrated in Table 1, IL-35 plays an important role in a variety of diseases. Furthermore, research from our group has identified that IL-35-expressing Tregs also play a central role in mediating tolerance in transplantation tolerance, immune responses to non-inherited maternal antigens (NIMAs), and prostate cancer [Ref. (23); and Olson et al., unpublished results], which we also found were dependent on the expression of CTLA-4. Interestingly, when we examined collagen type V-specific regulatory responses, we did not find a role for IL-35 [contrary to results obtained from other groups (16, 34)] nor CTLA-4, though we did find that these responses relied heavily on TGF-β. This suggests that the regulatory responses we identified in the transplant, NIMA, and prostate cancer patients may have been relying on iTr35 cells, whereas the responses we identified to collagen V were nTregs, and suggests that potentially CTLA-4 and IL-35-dependency may be a technique that can be used to identify this population of inducible regulatory cells.

**Figure 2:**
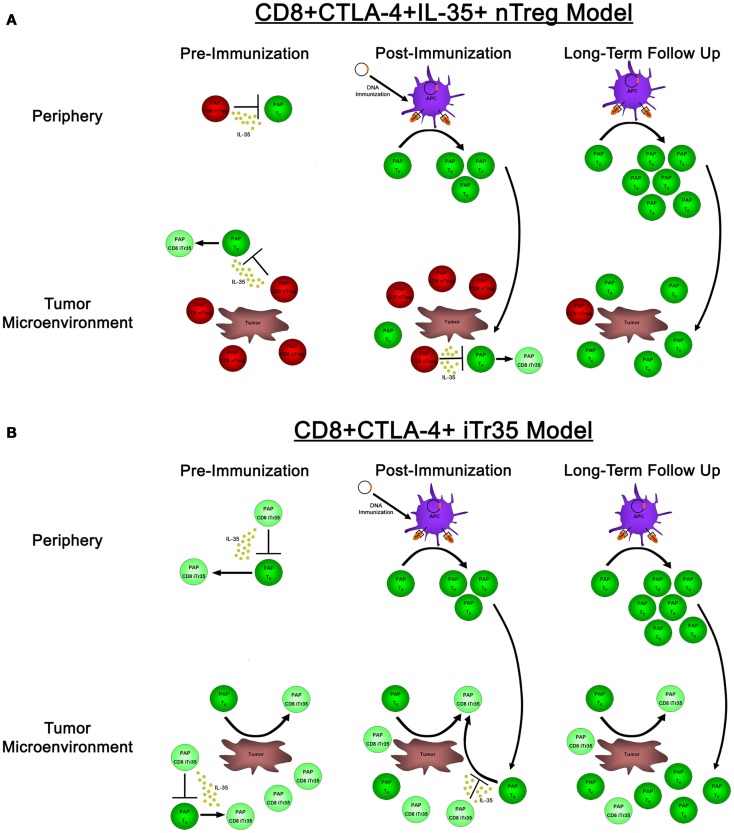
**The effects of natural versus induced PAP-specific CD8+ Tregs pre-immunization, post-immunization, and in long-term follow up**. **(A)** If the observed PAP-specific CD8+CTLA-4+ T cells represent a population of natural Tregs, pre-immunization samples (left panels) have PAP-specific CD8+ nTregs present (red cells) that utilize IL-35 to suppress the activity of PAP-specific effector cells (dark green) both in the periphery (top panels) as well as in the tumor microenvironment (bottom panels), as well as the ability to induce a population of IL-35-expressing Tregs (light green). Administration of a DNA vaccine encoding PAP leads to the presentation of PAP-derived epitopes on the surface of APCs immediately post-immunization (center panels), leading to the expansion of antigen-specific effector cells. However, these cells are in small numbers, and when they traffic to the tumor site, they are outnumbered by PAP-specific nTreg that are able to suppress their proliferation and effector functions. It is not until long-term follow up when these effector responses are able to outnumber antigen-specific nTreg, leading to the generation of productive anti-tumor immunity. **(B)** In a model where CD8+CTLA-4+ T cells represent a population of novel CD8+ iTr35 cells (light green cells), these iTregs would be able to convert effector cells (dark green) into additional iTreg through their expression of IL-35, thus propagating infectious tolerance to prevent the generation of productive anti-tumor immunity both pre-immunization as well as immediately post-immunization. This process would be predicted to continue until long-term follow up, when antigen-specific effectors could expand to a sufficient level to outnumber these iTreg responses, and potentially prevent the generation of induced antigen-specific Treg by promoting tumor destruction and a non-suppressive tumor microenvironment.

In our research evaluating the role of IL-35 expression in antigen-specific tolerance in prostate cancer patients, we identified CD8+CTLA-4+ IL-35-expressing T cells specific for the prostate tumor-associated antigen prostatic acid phosphatase (PAP), which were present in some patients with prostate cancer (23). Immediately following immunization with a DNA vaccine targeting PAP, these antigen-specific CD8+CTLA-4+ T cells prevented the detection of concurrent PAP-specific effector responses; however, in long-term follow ups, we found that PAP-specific effector responses could be detected in these individuals. These results raise questions regarding the nature of these CD8+CTLA-4+ IL-35-expressing regulatory cells; in particular, whether they represent a population of CD8+ T cells that have been induced in the periphery to gain IL-35 expression and suppressive activity, or alternatively if they are an antigen-specific thymus-derived population of nTregs. If these PAP-specific CD8+ regulatory responses represent a population of nTreg cells, their presence in the periphery and tumor microenvironment pre- and immediately post-immunization would suppress and prevent the detection of PAP-specific effector responses (as well as potentially induce the generation of IL-35-expressing Tregs), whereas the long-term generation and expansion of effector responses could eventually outnumber these suppressive responses and ultimately lead to the desired goal: the generation of productive, antigen-specific anti-tumor immunity (Figure 2A). Alternatively, if these PAP-specific CD8+ regulatory cells represent an induced population of CD8+ iTr35 cells, then their presence pre-immunization would be able to convert antigen-specific effector responses into additional regulatory responses (thus furthering the propagation of infectious tolerance) until extended period following immunization when effector responses overcome these regulatory responses, either by simply outnumbering regulatory CD8 T cells or by preventing the conversion of effector cells to regulatory cells through the generation of a non-immunosuppressive microenvironment (Figure 2B). Regardless, both of these models suggest that the goal of antigen-specific immunotherapies is not simply to generate effector responses, but rather to tip the balance between antigen-specific effector and regulatory responses toward productive anti-tumor immunity. Further research into these antigen-specific populations, how they are affected by antigen-specific vaccination, how they affect the generation of antigen-specific effector immune responses, and whether they have any predictive value toward the ultimate efficacy of anti-tumor vaccines, remains to be elucidated. While CD8+ Tregs are not as well studied as their CD4+ Treg counterparts, both CD8+ nTreg and iTreg have been identified and characterized, including reports in individuals with cancer (50). In our published studies, the reliance of PAP-specific CD8+CTLA-4+ T cells on IL-35 for mediating contact-independent suppression (with no identifiable role played by IL-10 nor TGF-β) suggests that these cells may be more akin to a population of CD8+ iTr35 cells which are dependent on IL-35 for contact-independent suppressive activity, as opposed to nTreg which are able to utilize multiple mechanisms of contact-independent suppression. Furthermore, our observation that the suppressive function of IL-35-expressing CD8+CTLA-4+ Treg is temporally regulated following antigen-specific immunization suggests that this population may be able to be modulated depending on the tumor microenvironment. However, additional research is required to determine how antigen-specific IL-35-expressing Treg are affected by antigen-specific immunization, as well as how these IL-35+ Treg responses are generated in tumor-bearing individuals.

To better characterize the generation and fate of iTr35 cells, it will be important to shed light onto the mechanisms that regulate the expression of IL-35 by Tregs. It is clear that expression of IL-35 by nTreg and iTreg requires activation, whether through inflammatory responses, non-specific stimulation of the T-cell receptor, or through encounter of antigen by antigen-specific Tregs (19, 22, 23). Additionally, it appears that Foxp3 does not directly play a role in the regulation of IL-35 expression, providing further evidence that IL-35 serves primarily as a potent mediator of suppression in induced regulatory populations rather than Foxp3+ nTregs (59). However, the regulation of Foxp3 does potentially open up new avenues of potential means of IL-35 regulation. Foxp3, along with other factors associated with Tregs such as CTLA-4, are specifically hypomethylated in nTreg cells, resulting in increased access to the transcriptional complex and higher expression levels compared to *T*_conv_ cells, where these sequences are preferentially hypermethylated (60, 61). Additionally, the expression of various cytokines has been shown to be epigenetically regulated, including IL-10 and TGF-β, which can in turn induce epigenetic changes that can lead to the generation of iTreg populations (62–65). This raises the possibility that the induction of IL-35 expression in iTr35 cells may be epigenetically regulated, which would permit the heritable transmission of IL-35 expression into subsequent progeny iTr35 cells while maintaining flexibility for altered expression levels based on the immune profile of the microenvironment. To date, epigenetic regulation of IL-35 expression has not been specifically evaluated – however, regions of the *IL-12p35* promoter have been shown to become methylated to regulate IL-12 expression by DCs (66) and *IL-12p35* intronic regions can become demethylated in non-activated Tregs (65). Additionally, the IL-12Rβ2 receptor has also been shown to be epigenetically regulated (67), suggesting that IL-35 could also be regulated using an epigenetic mechanism.

Many challenges also remain regarding the various populations of induced regulatory cells, and how these populations complement each other. Clearly there is a role for IL-10 in the generation of iTr35 cells, though the converse does not appear to be true, with IL-35 not appearing to play a central role in the generation of IL-10-secreting Tr1 or TGF-β-secreting Th3 populations. This may suggest that these populations may have distinct roles in the suppression of inflammatory immune responses. Alternatively, this could be a reflection of the redundancy of the immune system, having multiple layers of regulatory populations that can mediate similar effects, but that perhaps iTr35 cells represent more of a terminally differentiated regulatory cell that is mobilized when high levels of immunosuppression are required. While studying the differentiation pattern of this population raises significant experimental challenges, these studies will be essential to better understand the nature of T-cell functional plasticity, as well as how IL-35-expression fits into this process. Doing so will allow for the identification of methods that can be used to control and guide these regulatory responses to design effective therapies for cancer, autoimmunity, and tissue transplantation.

## Conflict of Interest Statement

The authors declare that the research was conducted in the absence of any commercial or financial relationships that could be construed as a potential conflict of interest.

## Abbreviations

APC, antigen-presenting cell; DC, dendritic cell; EAE, experimental autoimmune encephalitis; Ebi3, Epstein–Barr virus-induced gene 3; IL, interleukin; iTreg, induced regulatory T cell; NIMA, non-inherited maternal antigen; NK, natural killer; nTreg, natural regulatory T cell; PAP, prostatic acid phosphatase; TGF, transforming growth factor; Treg, regulatory T cell.
